# 6,6′-((Methylazanedyl)bis(methylene))bis(2,4-dimethylphenol) Induces Autophagic Associated Cell Death through mTOR-Mediated Autophagy in Lung Cancer

**DOI:** 10.3390/molecules27196230

**Published:** 2022-09-22

**Authors:** Nicharat Sriratanasak, Worawat Wattanathana, Pithi Chanvorachote

**Affiliations:** 1 Department of Pharmacology and Physiology, Faculty of Pharmaceutical Sciences, Bangkok 10330, Thailand; 2 Center of Excellence in Cancer Cell and Molecular Biology, Faculty of Pharmaceutical Sciences, Chulalongkorn University, Bangkok 10330, Thailand; 3 Department of Materials Engineering, Faculty of Engineering, Kasetsart University, Ladyao, Chatuchak, Bangkok 10900, Thailand

**Keywords:** benzoxazine dimers, mTOR inhibitor, apoptosis, non-small cell lung cancer, rapamycin

## Abstract

Autophagy is the multistep mechanism for the elimination of damaged organelles and misfolded proteins. This mechanism is preceded and may induce other program cell deaths such as apoptosis. This study unraveled the potential pharmacological effect of 24MD in inducing the autophagy of lung cancer cells. Results showed that 24MD was concomitant with autophagy induction, indicating by autophagosome staining and the induction of ATG5, ATG7 and ubiquitinated protein, p62 expression after 12-h treatment. LC3-I was strongly conversed to LC3-II, and p62 was downregulated after 24-h treatment. The apoptosis-inducing activity was found after 48-h treatment as indicated by annexin V-FITC/propidium iodide staining and the activation of caspase-3. From a mechanistic perspective, 24-h treatment of 24MD at 60 μM substantially downregulated p-mTOR. Meanwhile, p-PI3K and p-Akt were also suppressed by 24MD at concentrations of 80 and 100 μM, respectively. We further confirmed m-TOR-mediated autophagic activity by comparing the effect of 24MD with rapamycin, a potent standard mTOR1 inhibitor through Western blot and immunofluorescence assays. Although 24MD could not suppress p-mTOR as much as rapamycin, the combination of rapamycin and 24MD could increase the mTOR suppressive activity and LC3 activation. Changing the substituent groups (R groups) from dimethylphenol to ethylphenol in EMD or changing methylazanedyl to cyclohexylazanedyl in 24CD could only induce apoptosis activity but not autophagic inducing activity. We identified 24MD as a novel compound targeting autophagic cell death by affecting mTOR-mediated autophagy.

## 1. Introduction

Lung cancer is a primary public health issue worldwide. According to 2021 statistics, almost 25% of all cancer deaths are due to lung cancer [1]. Current chemotherapy focuses on apoptosis induction as a strategy for anticancer therapy [2]. Unfortunately, cancer cells can acquire resistance to apoptosis due to the dysregulation of anti-apoptotic or pro-apoptotic proteins [3,4]. For a decade, autophagy has been introduced as a backup strategy for cancer therapy [5]. Autophagy is a catabolic process in which intracellular components are engulfed and digested in autolysosome. Autophagy acts as a key cytoprotective mechanism by maintaining cellular homeostasis. However, this process may be activated to overcome cell death. “Autophagy-mediated cell death” is the mode when autophagy accompanies and induces other cell death modes, such as apoptotic cell death [6,7]. Autophagy may also promote apoptosis by degrading cell survival factors and anti-apoptotic proteins [8]. Moreover, the induction of autophagy is associated with p53-dependent apoptotic cell death [9]. Therefore, induction of autophagy-mediated cell death is believed to be a promising anticancer strategy.

Autophagy is shown to be tightly controlled by the mammalian target of rapamycin complex 1 (mTOR1), a downstream signaling in the phosphatidylinositol 3-kinase (PI3K)/Akt pathway. mTOR phosphorylates the ULK1 component to modulate phagophore formation. In membrane elongation, microtubule-associated protein 1 light chain 3 (LC3-I) is cleaved by ATG4 to form LC3-II before being conjugated with phosphatidyl ethanolamine by the ATG5-ATG12-ATG16 complex [10]. LC3 plays a crucial role in cargo selection and recruitment and therefore has been identified as a specific marker for autophagy. Ubiquitin-binding proteins, such as p62, serve as adaptors for targeted components to autophagic processes [11]. As a master regulator of autophagy, mTOR inhibitors have been studied in clinical trials for autophagic induction. Several mTOR inhibitors have been approved for cancer therapy [12], and some have shown beneficial effects of anti-aging and increasing lifespan [13]. Rapamycin is a selective mTOR1 inhibitor, and mTOR2 is partially inhibited after chronic rapamycin treatment [14]. This mTOR inhibitor is widely used as a standard in preclinical treatment [15].

c-Myc is identified as a major hallmark for several cellular biosyntheses, cell proliferation and cell survival [16]. The inactivation or inhibition of c-Myc can induce tumor regression by restoring of the normal cell checkpoint mechanisms or inducing of proliferation arrest, cellular senescence and apoptotic mechanisms [17,18]. c-Myc can be regulated by the Akt/mTOR signaling pathway [19]; and the inhibition of this signal transduction by mTOR inhibitor might affect c-Myc. In addition, c-Myc protein downregulation induces apoptosis and autophagy [20].

6,6′-((methylazanedyl)bis(methylene))bis(2,4-dimethylphenol) or 24MD is a benzoxazine dimer analog obtained from ring-opening reactions between dihydro-benzoxazines and phenols [21]. Previous studies have demonstrated the anticancer effect of another benzoxazine dimer analog, 2,2′-((methylazanedyl)bis(methylene))bis(4-ethylphenol) or EMD [22,23]. In the present work, we aim to explore the difference in anticancer activities when the substituent groups (R groups) on benzoxazine dimer in 24MD are changed. We aim to investigate the activity of 24MD on autophagy-mediate cell death in lung cancer cells and explored the key protein signal involved in pharmacology. Moreover, we identify specific chemical structure in mTOR inhibition activity through the comparison among 24MD, EMD, and 6,6′-((cyclohexylazanedy)bis(methylene))bis(2, 4-dimethylphenol) or 24CD.

## 2. Results

### 2.1. Effect of 24MD on Cytotoxicity and Apoptosis-Inducing Effect

**Figure 1:**
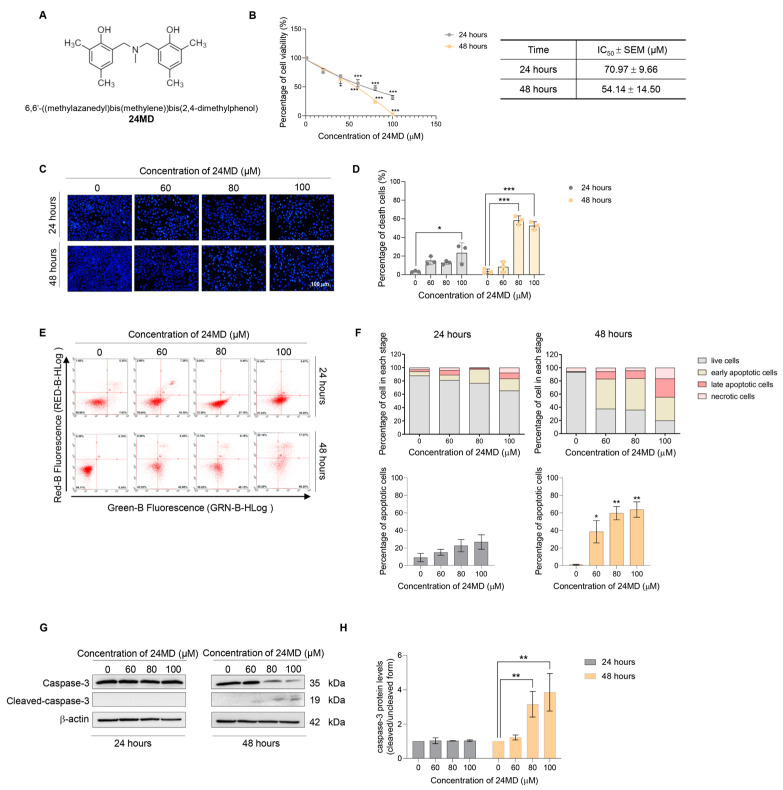
Effect of 24MD on cell viability and apoptosis inducing activity for 24 and 48 h of treatment. (**A**) the chemical structure of 24MD. (**B**) The MTT assay was performed to evaluate the cytotoxicity of 24MD. A549 cells were treated with various concentrations of 24MD (0–100 μM) for 24 and 48 h. The percentage of cell viability was calculated by comparison with non-treatment control. The half maximal inhibitory concentration (IC_50_) of 24MD at 24 and 48 h against A549 cells were estimated by using a linear regression equation. (**C**,**D**) Morphological changes of nucleus after treatment of 24MD (0–100 μM) for 24 and 48 h were examined by nuclear staining assay with Hoechst 33342. Fragmented chromatin of apoptotic cells was visualized with condensed blue fluorescence. The percentage of apoptotic cells was calculated. (**E**,**F**) A549 cells were treated with 24MD (0–100 μM) for 24 and 48 h before being subjected to evaluate apoptosis inducing activity by flow cytometry with annexin V FITC/propidium iodide (PI) staining. The percentage of cells in each stage and percentage of apoptotic cell death were demonstrated. (**G**,**H**) Key molecular protein of apoptosis inducing activity, caspase-3 was evaluated by Western blot analysis after 24MD (0–100 μM) treatment for 24 and 48 h. Densitometric analysis was performed, and the result was represented in relative protein levels when compared with non-treatment control at each time point. β-actin protein was measured to confirm equal loading in each sample. The data are demonstrated as mean ± SEM (*n* = 3) (* 0.01 ≤ *p* < 0.05, ** 0.001 ≤ *p* < 0.01 and *** *p* < 0.001 when compared with non-treatment control).

Human lung cancer cells, A549 were treated with various concentrations of 24MD (0–100 μM) for 24 and 48 h to elucidate the potential cytotoxic inducing activity of 24MD. Cell viability was then analyzed by MTT assay. 24MD significantly reduced cell viability compared with that in nontreatment control at each time point. The half maximal inhibitory concentration (IC_50_) was 70.97 ± 9.66 and 54.14 ± 14.50 µM at 24 and 48 h, respectively (Figure 1B).

Apoptosis is an important program to inhibit cancer progression and is characterized by cell membrane alteration and blebbing, chromatin condensation and DNA fragmentation [24]. Therefore, the morphologic changes of the nucleus were examined by a nuclear staining assay with Hoechst33342 to reveal apoptosis-inducing activity of 24MD. A549 cells were treated with various concentrations of 24MD (0–100 µM) for 24 and 48 h, then stained, and finally visualized under a fluorescence microscope. The condensed and/or fragmented nuclei were significantly observed at 100 µM 24MD after 24 h treatment and strongly increased at 80 µM 24MD after 48-h treatment compared with those in the nontreatment control (Figure 1C,D).

Flow cytometry with Annexin V FITC/PI was performed to confirm the apoptosis-inducing activity. The results revealed that treatment of 24MD for 24 h did not significantly increase the number of apoptotic cell deaths. By contrast, the apoptotic cell death was notably increased in 48 h 24MD treatment compared with that in the nontreatment control (Figure 1E,F). The expression of an essential apoptotic marker, caspase-3, was examined by Western blot analysis after treatment with various concentrations of 24MD (0–100 µM) for 24 and 48 h. Similar to the results of flow cytometry with Annexin V FITC/PI, the expression activated caspase-3 was significantly increased in the 48-h treatment but not in the 24-h treatment compared with that in the nontreatment control (Figure 1G,H).

### 2.2. 24 MD Induced Autophagy in Non-Small Cell Lung Cancer Cells

**Figure 2:**
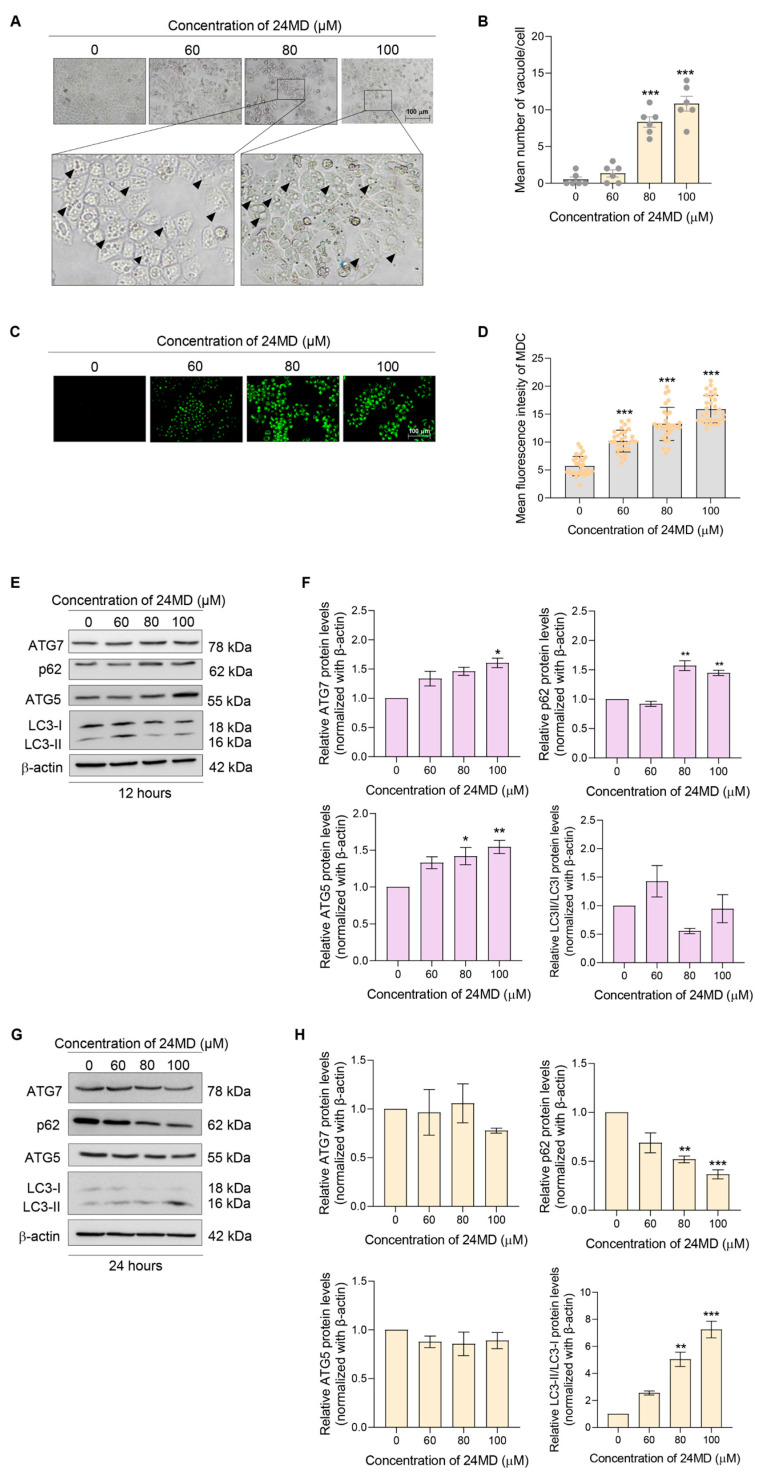
Effect of 24MD on autophagy induction and autophagic flux in A549 cells. (**A**,**B**) A549 cells were treated with 24MD (0–100 μM) for 24 h. The morphological changes of the A549 cells were visualized under a phase contrast microscope, and the number of vacuoles per cells were calculated. (**C**,**D**) A549 cells were stained with MDC after treatment with various concentrations of 24MD (0–100 μM) for 24 h and visualized under a fluorescence microscope. The green fluorescence of MDC represented the autophagic vacuole. The fluorescence intensity was measured, and the relative mean intensity was calculated. (**E**–**H**) Western blot analysis was performed to assess the autophagic markers, ATG5, ATG7, p62 and LC3B after 24MD treatment for 12 h and 24 h in A549 cells. β-actin protein was measured to confirm equal loading in each sample. The densitometry of each protein levels was calculated, and the results were demonstrated in relative protein levels. Data represent the mean ± SEM (*n* = 3) (* 0.01 ≤ *p* < 0.05, ** 0.001 ≤ *p* < 0.01 and *** *p* < 0.001 when compared with non-treatment control).

Autophagy is involved in the delivery of cytoplasmic cargoes for degradation and can be defined by the accumulation and increase of autophagic vacuoles in the cells [25]. Therefore, the cytoplasmic vacuoles were observed after treatment with various concentrations of 24MD (0–100 µM) for 24 h. The number of cytoplasmic vacuoles increased at 80–100 µM 24MD (Figure 2A,B). The A549 lung cancer cells were then treated and stained with monodansylcadaverine (MDC) to further identify the autolysosomes. The mean fluorescence intensity of MDC in a 24MD-treated group was significantly elevated compared with that in the nontreatment control (Figure 2C,D).

Western blot analysis was carried out to confirm the expression of autophagy-related marker proteins, such as LC3-I to II conversion, SQSTM1/p62, and many ATG proteins. A time-course experiment was also performed to evaluate the alteration of the proteins after the A549 cells were treated with various concentrations of 24MD for 12 and 24 h. At 12 h, 24MD notably upregulated SQSTM1/p62, ATG5 and ATG7 expression; however, LC3-I to II conversion was not significantly changed (Figure 2E,F). By contrast, after 24 h treatment, ATG5 and ATG7 were restored to the same levels as those in the nontreatment group. Meanwhile, LC3-I to II conversion was strongly elevated, and SQSTM1/p62 was remarkably decreased (Figure 2G,H).

### 2.3. 24 MD Mediated mTOR Signaling and Enhanced mTOR Inhibition

**Figure 3:**
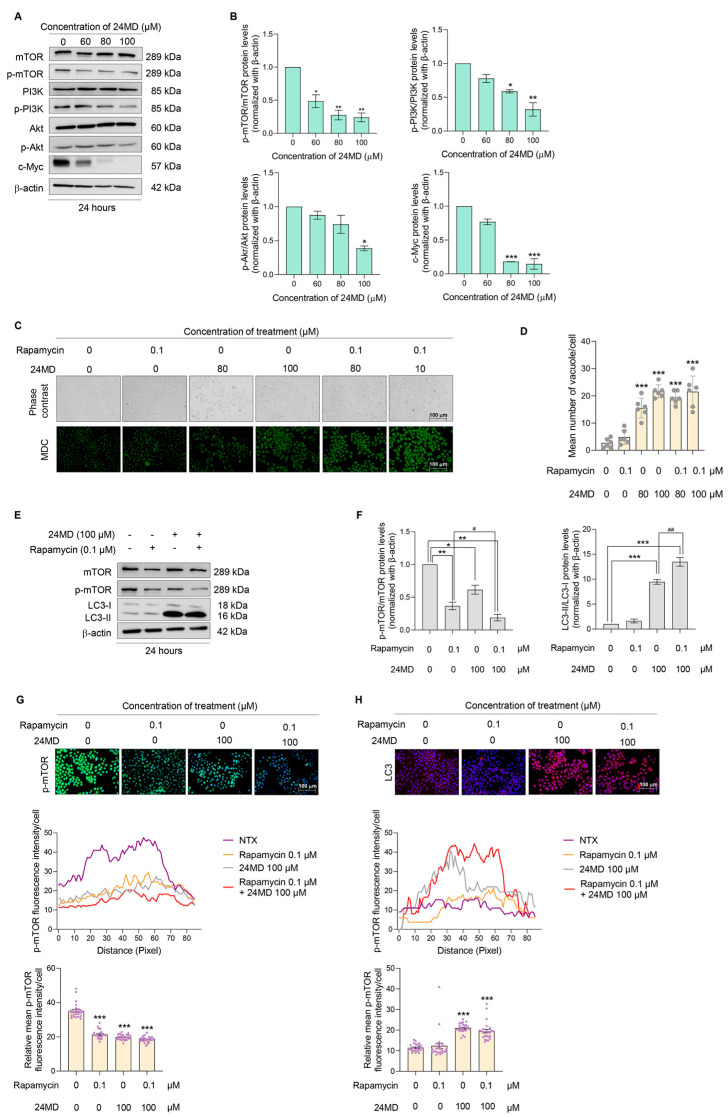
A549 cells were treated with 24MD (0–100 μM) for 24 h. (**A**,**B**) Western blot analysis was applied to evaluate the mTOR-mediated autophagy, which is the essential regulator. β-actin protein was evaluated to confirm equal loading of proteins in each sample. Densitometry of each protein levels were calculated, and the results were demonstrated in relative protein levels. (**C,D**) Rapamycin enhances the autophagic inducing effect of 24MD in A549 cells. The A549 cells were treated with various concentrations of 24MD (0–100 μM) with or without rapamycin at a concentration of 0.1 μM for 24 h. The fluorescence intensity of MDC was measured and the relative mean intensity was calculated. (**E**,**F**) Western blot analysis was performed to evaluate the effect of 24MD (0–100 μM) with or without rapamycin (0.1 μM) against mTOR and LC3B protein. β-actin protein was evaluated to confirm equal loading of protein in each sample. Densitometry of each protein levels was calculated, and the results were demonstrated in relative protein levels. (**G**,**H**) To confirm the effect of 24MD against mTOR-mediated autophagic cell death, the protein expression of p-mTOR and LC3B was evaluated by using an ICC assay. The fluorescence intensity per cell was measured, and the relative mean intensity per cell was calculated. Data represent the mean ± SEM (*n* = 3) (* 0.01 ≤ *p* < 0.05, ** 0.001 ≤ *p* < 0.01 and *** *p* < 0.001 when compared with non-treatment control) (# 0.01 ≤ *p* < 0.05, ## 0.001 ≤ *p* < 0.01 when compared with rapamycin treatment group or 24MD treatment group).

Autophagy is rigorously regulated by the PI3K/Akt/mTOR signaling pathway. Hence, the expression levels of key proteins, i.e., Akt and p-Akt, mTOR and p-mTOR, PI3K and p-PI3K and cell proliferation marker, c-Myc were monitored in the A549 lung cancer cells treated with various concentrations of 24MD (0–100 µM) for 24 h. The ratios of p-mTOR/mTOR, p-PI3K/PI3K and p-Akt/Akt were essentially downregulated by 24MD at concentrations of 60, 80 and 100 µM, respectively. Meanwhile c-Myc protein level was strongly suppressed (Figure 3A,B).

mTOR inhibition induces autophagosome formation. Rapamycin, a mTOR inhibitor, has been shown to induce autophagy and widely used for autophagy induction. Here, the induction of autophagic effect was first evaluated by MDC staining after 100 µM 24MD treatment with or without 0.1 µM rapamycin. The results exhibited that the combination of 24MD and rapamycin notably increased the mean fluorescence intensity compared with that in the nontreatment control but did not alter the intensity compared with that in the 24MD treatment alone (Figure 3C,D).

The ratio of p-mTOR/mTOR was revealed by Western blot analysis after treatment with rapamycin or 24MD alone or their combination to elucidate the effect against mTOR. The ratio was prominently diminished in all three treatment conditions. The combination treatment could remarkably decrease the p-mTOR/mTOR ratio compared with that in the rapamycin-treated group. Moreover, LC3-I to II conversion significantly changed in the 24MD and combination treatment groups compared with that in the nontreatment control. The combination of 24MD and rapamycin could notably elevate LC-I to II conversion compared with that in the 24MD-alone treatment group (Figure 3E,F).

Immunofluorescence assay was performed to confirm the expression of essential protein markers, p-mTOR and LC3. Similar to the finding of Western blot analysis, the protein expression of p-mTOR was highly suppressed and that of LC3 was increased (Figure 3G,H).

### 2.4. Cytotoxicity and mTOR- Inhibiting Activities of EMD and 24CD

**Figure 4:**
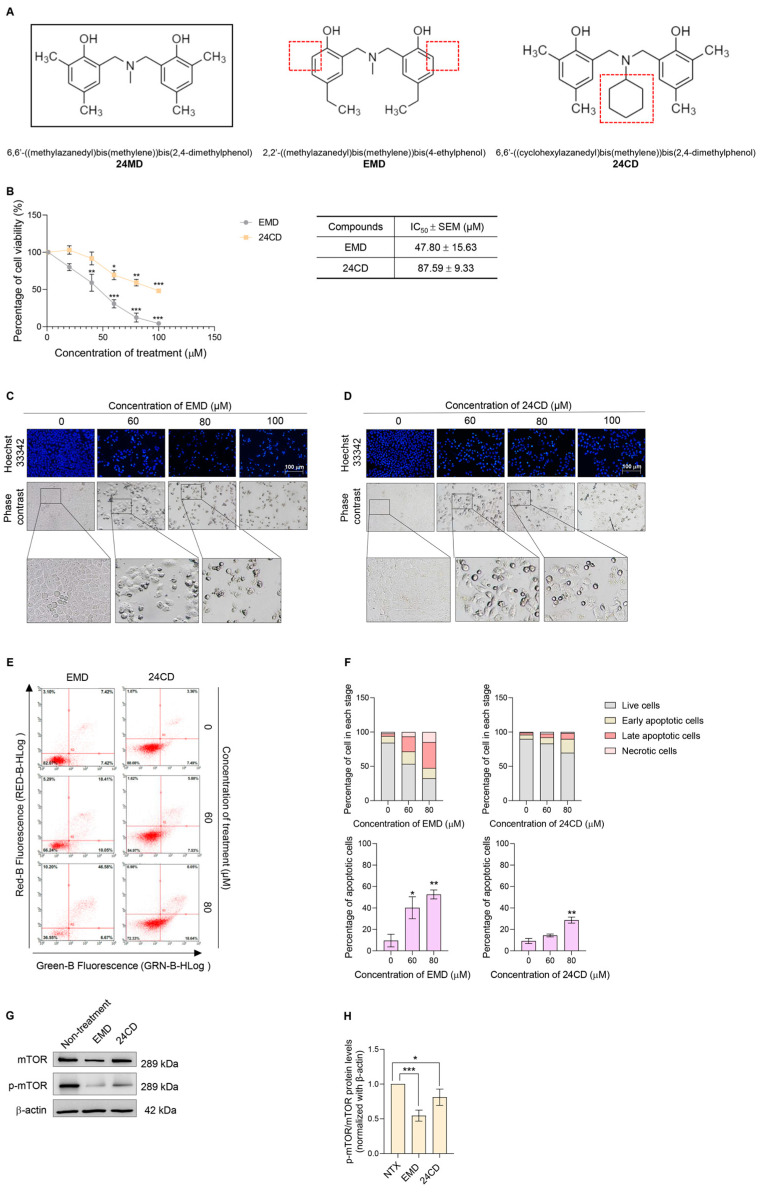
To demonstrate the structural related activity of 24MD, other benzoxazine dimer analogues, EMD and 24CD were applied for activity comparison. (**A**) Comparison of chemical structure between 24MD, EMD and 24CD. (**B**) The MTT assay was performed to evaluate the cytotoxicity of EMD and 24CD. A549 cells were treated with various concentrations of EMD and 24CD (0–100 μM) for 24 h. The percentage of cell viability was calculated by comparison with non-treatment control. The half maximal inhibitory concentration (IC_50_) of EMD and 24CD at 24 h against A549 cells were estimated by using linear regression equation. (**C**,**D**) Nuclear staining with Hoechst 33342 was performed to access morphological changes of nucleus after EMD and 24CD treatment (0–100 μM) for 24 h. The morphological changes of the A549 cells were visualized under phase contrast microscope. (**E**,**F**) A549 cells were treated with EMD and 24CD (0–80 μM) for 24 h before being subjected to evaluate apoptosis inducing activity by flow cytometry with annexin V FITC/propidium iodide (PI) staining. The percentage of cells in each stage and percentage of apoptotic cell death were demonstrated. (**G**,**H**) The effect of EMD and 24CD against mTOR and p-mTOR was examined by Western blot analysis. β-actin protein was evaluated to confirm the equal loading of protein in each sample. Densitometry of each protein levels was calculated, and the results were demonstrated in relative protein levels. Data represent the mean ± SEM (*n* = 3) (* 0.01 ≤ *p* < 0.05, ** 0.001 ≤ *p* < 0.01 and *** *p* < 0.001 when compared with non-treatment control).

We further question whether the presence of a different substituent group (R-group) on benzoxazine dimer might affect the autophagic induction. 2,2′-((methylazanedyl)bis(methylene))bis(4-ethylphenol) or EMD is an anticancer compound that targets c-Myc protein [23] and integrin β3 [22]; however, its autophagic induction effect has not been reported. Another benzoxazine dimer analog, 6,6′-((cyclohexylazanedyl)bis(methylene))bis(2, 4-dimethylphenol) or 24CD was also introduced in the comparison experiment. EMD has 4,ethylphenol substitution on benzine ring instead of the 2,4 dimethylphenol in 24MD. Meanwhile, 24CD has N-methyl substituent as a replacement for the N-cyclohexyl substituent (Figure 4A).

The cytotoxicity of EMD and 24CD in A549 lung cancer cells was determined by MTT assay. The A549 cells were treated with various concentrations of EMD or 24CD (0–100 µM) for 24 h. EMD and 24CD significantly decreased cell viability with IC_50_ at 47.80 ± 15.63 and 87.59 ± 9.33 µM, respectively. A nuclear staining assay with Hoechst33342 was applied to explore nuclear morphological changes after EMD or 24CD treatment (Figure 4C,D). Phase contrast results revealed no vacuole formation in EMD- and 24CD-treated groups (Figure 4C,D).

We further confirmed the apoptosis-inducing activity of EMD or 24CD by flow cytometry with Annexin V FITC/PI. A549 cells were treated with EMD or 24CD at concentrations of 0–80 µM for 24 h. Apoptotic cell death significantly induced apoptotic cell death by EMD starting at a concentration of 60 µM and 24CD starting at a concentration of 80 µM (Figure 4E,F).

A549 lung cancer cells were treated with IC_50_ concentrations of EMD or 24CD for 24 h to examine their effect against mTOR protein. Western blot analysis was performed to evaluate the p-mTOR/mTOR protein ratio. EMD demonstrated a stronger effect against a p-mTOR/mTOR ratio than 24CD (Figure 4G,H).

## 3. Discussion

Apoptosis and autophagy are both anticancer pathways. At present, most first-line chemotherapy for lung cancer, such as platinum-based compounds, commonly result in apoptotic cell death [26]. Unfortunately, only 20–30% of patients with lung cancer respond to conventional chemotherapy [27]. This poor rate is essentially caused by defective apoptosis [3]. Autophagy is identified as a tumor suppressor pathway because it manipulates the degradation of oncogenic molecules [28]. Apoptosis and autophagy could occur in the same cell. The cells which commence apoptotic cell death may not exhibit autophagy due to the caspase-mediated cleavage of kay autophagic proteins [29]. By contrast, autophagy usually appears before apoptosis. Autophagy induction generates caspase-8 and activates the effector caspase-3 [30]. Therefore, these two-cell death mechanisms are capable of a crosstalk effect. Therefore, autophagy-mediated apoptosis might be a promising strategy for lung cancer therapy.

Autophagy is a cellular process that maintains intracellular homeostasis. In general, autophagy degrades dysfunctional cytoplasmic components, including oncoproteins by delivering them into the autolysosome [31]. This process is identified as a pro-survival mechanism for chemotherapy. Autophagy activation could mediate the acquired resistance phenotype [32]. However, this mechanism is a double-edged sword. It can play a role in autophagic cell death (ACD) or in autophagy-mediated cell death [33]. Several autophagic proteins exhibit crosstalk regulation between apoptosis and autophagy. Beclin-1 is recognized as an autophagy effector regulating phagophore formation. Its elevated expression may induce the release of Bak/Bax, a pro-apoptotic protein, from Bcl-2, an anti-apoptotic protein, to promote apoptosis [34]. ATG12-ATG5-ATG7 complex is essential for membrane elongation of autophagosome. ATG12 upregulation may also inhibit Mcl-1 activities leading to apoptosis induction [35]. Truncated ATG5, which is cleaved by calpain, could activate Bcl-XL and then induce cytochrome c release and caspase cascade [36]. As a backup cell death mechanism [5], autophagy may offer a new avenue to support cancer therapy in patients with defective apoptosis. The PI3K/Akt/mTOR signaling pathway mainly regulates autophagy mechanism. mTOR1 is defined as a key regulator for cell growth, metabolism, and autophagy, and its inhibition results in autophagy-mediated cell death [37,38,39]. The mTOR complex inhibits autophagy by phosphorylating and inactivating of ULK1, an initiator of autophagic mechanism [40,41]. Consistently, we found that 24MD exerts its autophagy-mediated cell death induction via the suppression of the PI3K/Akt/mTOR signaling pathway.

24MD presented cytotoxicity in A549 lung cancer cells and significantly induced apoptosis cell death after treatment for 48 h as indicated by the result of flow cytometry with Annexin V FITC/PI and the caspase-3 activation (Figure 1). Meanwhile, the A549 cells exhibited numerous vacuoles after 24MD treatment at 80–100 µM for 24 h as evidenced by the escalation of MDC fluorescence intensity in treatment cells (Figure 2A–D). The treatment of 24MD in A549 lung cancer cells for 48 h demonstrated that most of the cells have already undergone apoptosis cell death. The apoptosis process activates the caspases, the enzymes that degrade protein components of cells. Therefore, treatment for 48 h might decrease the up-stream protein signaling and autophagic markers due to caspase-mediated protein cleavage. Therefore, we use the early time points for evaluation of up-stream signaling. Protein analysis was performed to reveal the occurrence of autophagic flux. The key markers of autophagic induction such as ATG5, ATG7 and p62 were significantly upregulated after 12-h 24MD treatment, but ATG5 and ATG7 were restored in the next 12 h (Figure 2E,F). LC3-I to II conversion strongly increased after the 24-h treatment of 24MD at 80–100 µM, and this result was coincidental with p62 reduction (Figure 2G,H). This phenomenon might be attributed to the degradation phase of autophagosome where p62 itself is degraded [42,43].

The treatment for 12 h causes an early stage of autophagic induction, which is represented in Figure 2E. The LC3I to II conversion was not significantly different at 12 h. Therefore, evaluation at 24 h where the autophagy was obviously observed and LC3I to II conversion was steady (Figure 2A–D,G,H) might suitably indicate the autophagy. Considering that the PI3K/Akt/mTOR axis is a main pathway involved in autophagic induction, we further evaluated the effect of 24MD against this important pathway. The results indicated that the main mechanism of 24MD in mediating autophagy was through mTOR suppression. p-mTOR protein level began to decrease after 24-h treatment of 60 µM 24MD, followed by the inhibition of p-PI3K and p-Akt at 80 and 100 µM 24MD, respectively. Another key cell survival protein is c-Myc, the downstream signaling from PI3K/Akt/mTOR pathway. Its expression also dramatically decreased. Depletion of c-Myc is one of the significant factors in apoptosis induction [44,45] (Figure 3A,B). Rapamycin is a well-known selective mTOR inhibitor; however, long-term treatment with this drug also alters mTOR2 in some cell types [13]. The efficacy of 24MD and rapamycin combination treatment for A549 lung cancer cells was assessed. The results showed that the treatment with rapamycin at 0.1 µM for 24 h could not induce autophagy but significantly reduced p-mTOR compared with 24MD. The combination of rapamycin and 24MD had a stronger inhibition effect than rapamycin alone treatment (Figure 3C–H).

Other benzoxazine analogs, EMD and 24CD were evaluated for anticancer activity. EMD and 24CD also exhibited cytotoxicity against A549 lung cancer cells (Figure 4). Similar to a previous study [23], EMD but not 24CD could significantly induce apoptotic cell death (Figure 4C–F) at desired concentrations after 24-h treatment. Earlier chemical structure functional studies reported that EMD has the highest oxidative potential among the evaluated compounds that could generate the highest oxidative stress. Substitution with methyl groups at 2 and 4 positions on benzene ring in 24MD and 24CD provided these compounds a critical potential in stabilizing the cationic intermediates; the cation can be distributed to 2 and 4 positions on the benzene ring due to the effect of the hydroxyl group. The steric effect of substituents in 24CD molecules might promote the formation of intramolecular hydrogen bond (HB) [21], resulting in the subtraction of a couple of donor/acceptor functional motifs. The intramolecular HB could increase lipophilicity and enhance membrane permeability [46] while reducing aqueous solubility [47]. The molecules with intermolecular HB cannot interact with the solvent water molecules via H bonding because their polar functional groups are involved in an intramolecular interaction [48]. Therefore, 24CD precipitation occurred at concentration of 80 µM onward after being diluted with culture medium. The strong ROS induction might bypass autophagic mechanism to apoptotic cell death by inducing the expression of pro-apoptotic proteins such as caspase-3 activity [49]. Therefore, we could not observe autophagic induction in the EMD treatment. 24MD might only overbalance the oxidative stress after 24 h, resulting in autophagic induction. Apoptotic cell death was observed after continuing the treatment to 48 h. Considering that 24MD demonstrated significant p-mTOR reduction, the key regulator of autophagic and cell survival mechanism, we further evaluated the effect of these three benzoxazine dimer analogs against the mTOR protein. We only performed at the same time as we detected the alteration of p-mTOR protein expression in 24MD treatment (Figure 3A,B). At IC_50_ concentration, EMD demonstrated the largest effect against p-mTOR among these three analogs. Meanwhile, 24CD had no effect against the p-mTOR/mTOR ratio. Therefore, the different substituents on the benzoxazine dimer might relate to the cytotoxic activity and cellular molecular signal. Further work should focus on the effect of chemical structure on interested proteins. Structural-activity relationships and chemical properties that might affect the efficiency of the compound as an anticancer therapy must be investigated.

## 4. Materials and Methods

### 4.1. Reagents and Antibodies

Dulbecco’s Modified Eagle’s Medium (DMEM) medium, fetal bovine serum (FBS), antibiotic-antimycotic, L-glutamine supplement, phosphate-buffered saline (PBS), and 0.25% trypsin-EDTA were obtained from Gibco (Grand Island, NY, USA). 3-(4,5-dimethylthiazol-2-yl)-2,5-diphenyltetrazolium bromide (MTT) and monodansylcadaverine (MDC) were acquired from Invitrogen, Thermo Fisher (Waltham, MA, USA). Dimethyl sulfoxide (DMSO) and Hoechst 33342 were purchased from Sigma Aldrich, Co. (St. Louis, MO, USA). Bovine serum albumin (BSA) and skim milk powder were obtained from Merck Millipore (HES, Germany). The primary antibodies, c-Myc (#5605), SQSTM1/p62 (#2947), p53 (#2527), Akt (#9272), phosphorylated Akt or p-Akt (#4046), caspase-3 (#2662), mTOR (#9283), p-mTOR (#5536), ATG5 (#12994), ATG7 (#8558), PI3K (#4292), p-PI3K (#4228), LC3B (#3868), β-actin (#8457) and the secondary antibodies for Western blot analysis, anti-rabbit IgG (#7074) were acquired from Cell Signaling Technology (Danvers, MA, USA). The secondary antibodies for immunocytochemistry, Alexa Fluor 488 goat anti-rabbit IgG (A11034) and Alexa Fluor 594 goat anti-rabbit IgG (A11037) were obtained from Invitrogen, Thermo Fisher (Waltham, MA, USA). Paraformaldehyde and 2,4-dimethylphenol were purchased from Sigma Aldrich, while methylamine (40% *w/v* in water) and anhydrous sodium sulfate were supplied from Merck. Cyclohexylamine was bought from Alfa Aesar. Sodium hydroxide and 4-ethylphenol were obtained from Ajax Finechem and Fluka Chemicals, respectively. The solvents such as dichloromethane, diethyl ether, propan-2-ol, and dioxane were received from RCI Labscan. All chemicals were analytical grade and used as received. EMD, 24MD and 24CD were delivered from the Department of Materials Engineering, Faculty of Engineering, Kasetsart University, Thailand.

### 4.2. Synthesis of EMD, 24MD and 24CD

**Figure 5:**
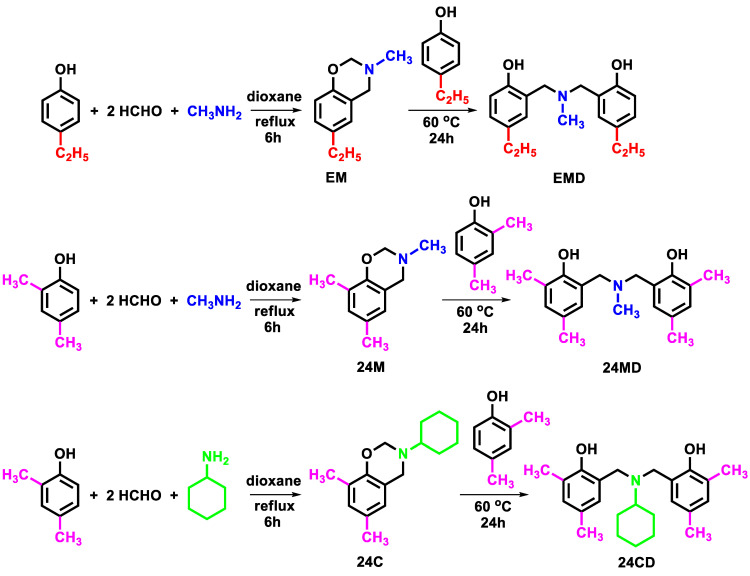
*N,N*-bis(2-hydroxybenzyl) alkylamine derivatives used in this work.

Three *N,N*-bis(2-hydroxybenzyl) alkylamines, namely 2,2′-(methylazanediyl)bis(methylene)bis(4-ethylphenol) (EMD), 6,6′-(methylazanediyl)bis(methylene)bis(2,4-dimethylphenol) or (24MD), and 6,6′-(cyclohexylazanediyl)bis(methylene)bis(2,4-dimethylphenol) or (24CD), were synthesized according to the reactions illustrated in Figure 5 [21,50,51,52,53]. The first step was a one-pot Mannich reaction to form benzoxazine heterocycles, and the second step was a ring-opening reaction of the benzoxazines and phenols. For the first step, three starting materials, phenol (4-ethylphenol or 2,4-dimethylphenol), formaldehyde, and amine (methylamine or cyclohexylamine) with the molar ratio of 1:2:1, were mixed with dioxane in a round bottom flask [54,55]. The mixture was then refluxed for 6 h for the reaction to be completed, resulting in the clear yellow solution. The dioxane solvent was removed using a rotary evaporator, and dichloromethane was then added to the mixture. The obtained solution was washed with 3N NaOH solution and deionized water to remove impurities. The dichloromethane layer was separated from the aqueous layer using a separatory funnel and was then dried with anhydrous sodium sulfate to obtain a pure benzoxazine product (EM, 24M, and 24C) as a sticky brown liquid. Next, the equimolar amount of phenol (4-ethylphenol or 2,4-dimethylphenol) was added to the obtained benzoxazine (EM, 24M, or 24C) without any other solvent and then heated at 60 °C for 24 h. The obtained product was washed with diethyl ether to remove impurities and to precipitate off the white precipitate of the desired product (EMD, 24MD, and 24CD). Further purification was performed by recrystallization of the white precipitate in propan-2-ol. The spectra data of the synthesized compounds were demonstrated in supporting information (Figures S1–S9).

### 4.3. Preparation of EMD, 24MD and 24CD Stock Solution

EMD, 24MD and 24CD were prepared as a 40 mM master stock solution by dissolving in DMSO and then diluted to 4, 8, 12, 16, and 20-mM stock solutions. All of the stock solutions were stored at −20 °C and were freshly diluted 200 times with DMEM completed medium to the required concentrations before treatment. The final concentrations of DMSO in all treatment conditions were less than 0.5% *v/v*.

### 4.4. Cell Line and Culture

The non-small cell lung cancer cell line A549 (ATCC^®^ CCL-185™, RRID: CVCL_0023™) was cultured in 10% FBS DMEM with 1% penicillin and streptomycin. The A549 lung cancer cells were stored at 37 °C in a humidified incubator of 5% carbon dioxide. The A549 lung cancer cells were used at about 75% confluence.

### 4.5. Cell Viability

For cytotoxicity activity testing, 1.5 × 10^4^ cells/well were seeded onto 96-well plates and incubated overnight. Then, A549 lung cancer cells were treated with various concentrations of EMD, 24MD or 24CD for 24 h at 37 °C and analyzed using the 3-(4,5-dimethylthiazol-2-yl)-2,5-diphenyltetrazolium bromide (MTT) assay according to the manufacturer’s protocol (Sigma-Aldrich). The absorbance of formazan was measured at 570 nm by the VICTOR^3^ Multilabel Plate Reader (PerkinElmer, CT, USA). To calculate cell viability, the absorbance of the treated cells was divided by that of nontreated cells and is reported as a percentage.

### 4.6. Nuclear Staining Assay

This assay reveals the morphological changing of nucleus in apoptosis inducting activity. The A549 lung cancer cells were stained with Hoechst33342 for 30 min at 37 °C after treatment with various concentrations of 24MD, EMD or 24CD for 24 h. After that, they were visualized and captured the image under a fluorescence microscope (Nikon ECLIPSE Ts2, Nikon, Tokyo, Japan). Results are reported as a percentage of apoptotic cells.

### 4.7. Flow Cytometry with Annexin V FITC/Propidium Iodide (PI)

This method was applied for examination of apoptotic cell death. The A549 lung cancer cells were treated with various concentrations of 24MD, EMD or 24CD for 24 and 48 h. Then, they were subjected and incubated with Annexin V FITC and PI for 15 min in the dark at room temperature. The A549 lung cancer cells were analyzed by guavaCyte^TM^ flow cytometry systems (guavasoft^TM^ Software version 3.3).

### 4.8. Western Blot Analysis

After treatment with decided condition, the A549 lung cancer cells were collected and incubated with RIPA lysis buffer which contains NaCl 150 mM, Tris-HCl pH 7.6 25 mM, 1% sodium deoxycholate, 1% NP-40, 0.1% SDS for 30 min at 4 °C. The lysates were collected and their protein content were determined using a BCA protein assay kit (Pierce Biotechnology, Rockford, IL, USA). An equivalent number of proteins from each sample were separated by SDS-PAGE and transferred to 0.2 μm polyvinylidene difluoride (PVDF) membranes (Bio-Rad). The separating blots were blocked with 5% skim milk in TBST (Tris-buffer saline with 0.1% tween containing NaCl 125 mM, Tris-HCl pH 7.5 25 mM and 0.1% tween 20) for 2 h and incubated with primary antibody overnight at 4°C. Secondary antibodies were incubated for 2 h at room temperature after being washed by TBST three times. Finally, the protein bands were detected using chemiluminescence substrate and exposed by Chemiluminescent ImageQuant LAS4000. Protein bands were analyzed using ImageJ software (version 1.52, National Institutes of Health, Bethesda, MD, USA).

### 4.9. Monodansylcadaverine (MDC) Staining

This assay was applied to assess the autophagic induction where the autophagic vacuoles are labeled by MDC. The A549 lung cancer cells were treated with decided conditions. Then, they were stained with 0.05 mM MDC for 30 min at 37 °C. After incubation, the A549 lung cancer cells were visualized by a fluorescence microscope (Nikon ECLIPSE Ts2).

### 4.10. Immunofluorescence

The A549 lung cancer cells were seeded at concentration of 8000 cells/well and cultured overnight. After that, the supernatant was removed and the A549 lung cancer cells were fixed with 4% paraformaldehyde for 15 min at room temperature or ice-cold methanol for 10 min at 4 °C. They were permeabilized with 0.5% Triton-X 100 in 10% FBS PBS for 5 min (no need to permeabilize in methanol fixation), followed by blocking the non-specific protein with 10% FBS in 0.1% Triton-X PBS for 1 h at room temperature. The primary antibodies were diluted to 1:50 *v/v* for p-mTOR and 1:200 for LC3 and incubated with the A549 lung cancer cells at 4 °C overnight. Later, the A549 lung cancer cells were incubated with secondary antibodies at a concentration of 1:500 *v/v* for 1 h before being stained with Hoechst 33342 for 15 min. Glycerol at concentration of 70% *v/v* was added. Then, the images were captured under a fluorescence microscope (Nikon ECLIPSE Ts2). The results were demonstrated in relative mean fluorescence intensity per cell.

### 4.11. Statistical Analysis

The results are presented as mean ± SEM of at least 3 independent measurements. Multiple comparisons for statistically significant differences between multiple groups were performed by ANOVA with a Scheffe post hoc test. For two-sample comparison, a one-sample *t*-test was calculated by an SPSS software program version 28 (SPSS Inc., Chicago, IL, USA). Statistical significance was considered at *p* < 0.05. GraphPad Prism 9 was used for creating graphs in all experiments.

## 5. Conclusions 

**Figure 6:**
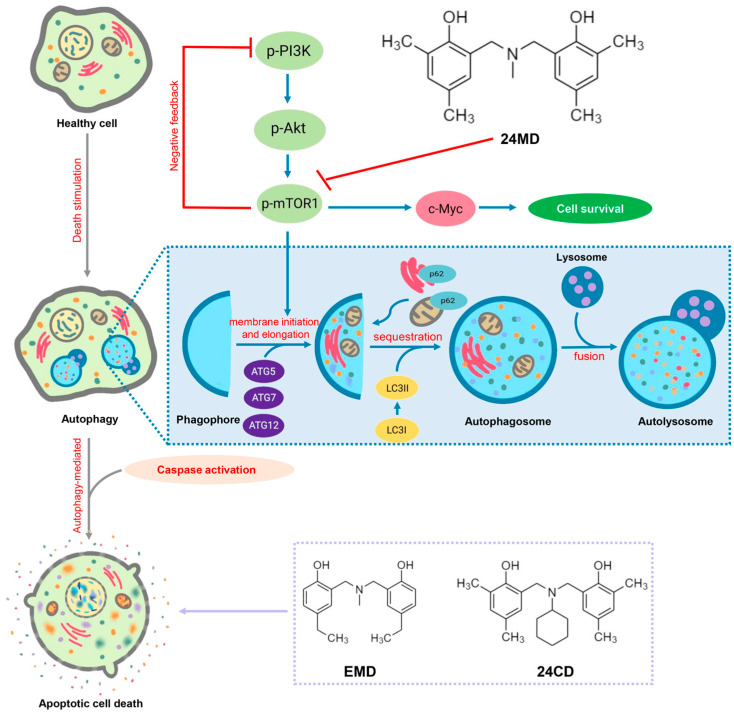
Autophagy is a multi-step catabolic process which controls cellular homeostasis and response to stress stimulation. As mTOR is the main autophagic suppressor, the type I PI3K-Akt-mTOR signaling which plays an important role is inhibited under the autophagic induction. The phagophore is generated, while ATG5-ATG12 associated with ATG7 is activated for vesicle elongation and completion. The other key marker which is involved in this step is LC3. LC3I is transformed to an autophagic-vesicle associated form LC3II. This cleaved form is designated as a marker of autophagy. The vesicle engulfs the cytosolic material which is recruited by a cargo receptor such as SQSTM1/p62. Then, a maturated autophagosome fuses with lysosomes to become autolysosomes. The engulfed material is degraded by acidic hydrolases and lysosomal enzymes. Autophagic-mediated apoptosis cell death is a process in which the autophagic induced apoptosis mechanism through degradation of the anti-apoptotic protein leads to activating the caspase cascade. 24MD could enhance autophagic-mediated apoptosis cell death by inhibiting mTOR signaling. However, the different substituted groups, EMD and 24CD, could only demonstrate the apoptosis inducing activity.

In conclusion, 24MD with benzoxazine dimer analogs can induce both ACD and apoptosis in non-small cell lung cancers (NSCLCs) by targeting mTOR suppression. This effect is the novel mechanism of action of 24MD compound in shifting survival role of autophagy toward autophagy-associated cell death. The evidence from this study may support further investigation on this lead compound for anticancer approaches and is beneficial for improving the response to conventional drugs (Figure 6).

## Data Availability

Data are contained within the article.

## Supplementary Materials

The following supporting information can be downloaded at: https://www.mdpi.com/article/10.3390/molecules27196230/s1, Figure S1: Fourier-transform infrared spectrum of EMD, Figure S2: ^1^H-NMR spectrum of EMD, Figure S3: ^13^C-NMR spectrum of EMD, Figure S4: Fourier-transform infrared spectrum of 24MD, Figure S5: ^1^H-NMR spectrum of 24MD, Figure S6: ^13^C-NMR spectrum of 24MD, Figure S7: Fourier-transform infrared spectrum of ECD, Figure S8: ^1^H-NMR spectrum of ECD, Figure S9: ^13^C-NMR spectrum of ECD.

## Sample Availability

Samples of the compounds EMD, 24MD and 24CD are available from the authors.

## Abbreviations

EMD	2,2′-(methylazanediyl)bis(methylene)bis(4-ethylphenol)
24MD	6,6′-(methylazanediyl)bis(methylene)bis(2,4-dimethylphenol)
24CD	6,6′-(cyclohexylazanediyl)bis(methylene)bis(2,4-dimethylphenol)
NSCLC	non-small cell lung cancer
ATG	autophagy related protein
LC3	microtubule-associated protein 1 light chain 3
Akt	protein kinase B
p-Akt	phospho-Protein kinase B
mTOR	mammalian target of rapamycin
PI3K	Phosphoinositide 3-kinases
ULK	Unc-51-like kinase
MTT	3-(4,5-dimethylthiazol-2-yl)-2,5-diphenyltetrazolium bromide
MDC	monodansylcadaverine
DMSO	dimethyl sulfoxide
NP40	nonyl phenoxypolyethoxylethanol

## References

[1] Siegel R.L., Miller K.D., Fuchs H.E., Jemal A. (2021). Cancer Statistics, 2021. CA Cancer J. Clin..

[2] Pistritto G., Trisciuoglio D., Ceci C., Garufi A., D’Orazi G. (2016). Apoptosis as anticancer mechanism: Function and dysfunction of its modulators and targeted therapeutic strategies. Aging.

[3] Fulda S. (2009). Tumor resistance to apoptosis. Int. J. Cancer.

[4] Igney F.H., Krammer P.H. (2002). Death and anti-death: Tumour resistance to apoptosis. Nat. Rev. Cancer.

[5] Fan Y.J., Zong W.X. (2013). The cellular decision between apoptosis and autophagy. Chin. J. Cancer.

[6] Jung S., Jeong H., Yu S.W. (2020). Autophagy as a decisive process for cell death. Exp. Mol. Med..

[7] Shimizu S., Nakao K., Minato N., Uemoto S. (2015). Autophagic Cell Death and Cancer Chemotherapeutics. Innovative Medicine: Basic Research and Development.

[8] Doherty J., Baehrecke E.H. (2018). Life, death and autophagy. Nat. Cell Biol..

[9] Wang S., Wang K., Zhang C., Zhang W., Xu Q., Wang Y., Zhang Y., Li Y., Zhang Y., Zhu H. (2017). Overaccumulation of p53-mediated autophagy protects against betulinic acid-induced apoptotic cell death in colorectal cancer cells. Cell Death Dis..

[10] Lippai M., Szatmari Z. (2017). Autophagy-from molecular mechanisms to clinical relevance. Cell Biol. Toxicol..

[11] Fougeray S., Pallet N. (2015). Mechanisms and biological functions of autophagy in diseased and ageing kidneys. Nat. Rev. Nephrol..

[12] Kim Y.C., Guan K.L. (2015). mTOR: A pharmacologic target for autophagy regulation. J. Clin. Investig..

[13] Li J., Kim S.G., Blenis J. (2014). Rapamycin: One drug, many effects. Cell Metab..

[14] Waldner M., Fantus D., Solari M., Thomson A.W. (2016). New perspectives on mTOR inhibitors (rapamycin, rapalogs and TORKinibs) in transplantation. Br. J. Clin. Pharm..

[15] Klionsky D.J., Abdel-Aziz A.K., Abdelfatah S., Abdellatif M., Abdoli A., Abel S., Abeliovich H., Abildgaard M.H., Abudu Y.P., Acevedo-Arozena A. (2021). Guidelines for the use and interpretation of assays for monitoring autophagy (4th edition)(1). Autophagy.

[16] Pelengaris S., Khan M., Evan G. (2002). c-MYC: More than just a matter of life and death. Nat. Rev. Cancer.

[17] Felsher D.W., Bishop J.M. (1999). Reversible tumorigenesis by MYC in hematopoietic lineages. Mol. Cell.

[18] Gabay M., Li Y., Felsher D.W. (2014). MYC activation is a hallmark of cancer initiation and maintenance. Cold Spring Harb. Perspect. Med..

[19] Chang T.M., Shan Y.S., Chu P.Y., Jiang S.S., Hung W.C., Chen Y.L., Tu H.C., Lin H.Y., Tsai H.J., Chen L.T. (2017). The regulatory role of aberrant Phosphatase and Tensin Homologue and Liver Kinase B1 on AKT/mTOR/c-Myc axis in pancreatic neuroendocrine tumors. Oncotarget.

[20] Hwang S.-K., Jeong Y.-J., Shin J.-M., Magae J., Kim C.-H., Chang Y.-C. (2018). MAC inhibits c-Myc and induces autophagy by downregulation of CIP2A in leukemia cells. Mol. Cell. Toxicol..

[21] Suetrong N., Chansaenpak K., Impeng S., Pinyou P., Blay V., Blay-Roger R., Lisnund S., Kanjanaboos P., Hanlumyuang Y., Wannapaiboon S. (2021). Influences of Chemical Functionalities on Crystal Structures and Electrochemical Properties of Dihydro-benzoxazine Dimer Derivatives. Crystals.

[22] Sriratanasak N., Nonpanya N., Wattanathana W., Chanvorachote P. (2020). Benzoxazine Dimer Analogue Targets Integrin beta3 in Lung Cancer Cells and Suppresses Anoikis Resistance and Migration. Anticancer Res..

[23] Sriratanasak N., Petsri K., Laobuthee A., Wattanathana W., Vinayanuwattikun C., Luanpitpong S., Chanvorachote P. (2020). Novel c-Myc-Targeting Compound N, N-Bis (5-Ethyl-2-Hydroxybenzyl) Methylamine for Mediated c-Myc Ubiquitin-Proteasomal Degradation in Lung Cancer Cells. Mol. Pharm..

[24] Su Z., Yang Z., Xu Y., Chen Y., Yu Q. (2015). Apoptosis, autophagy, necroptosis, and cancer metastasis. Mol. Cancer.

[25] Kroemer G., Levine B. (2008). Autophagic cell death: The story of a misnomer. Nat. Rev. Mol. Cell Biol..

[26] Bironzo P., Di Maio M. (2018). A review of guidelines for lung cancer. J. Thorac. Dis..

[27] Scheff R.J., Schneider B.J. (2013). Non-small-cell lung cancer: Treatment of late stage disease: Chemotherapeutics and new frontiers. Semin. Interv. Radiol..

[28] Su M., Mei Y., Sinha S. (2013). Role of the Crosstalk between Autophagy and Apoptosis in Cancer. J. Oncol..

[29] Marino G., Niso-Santano M., Baehrecke E.H., Kroemer G. (2014). Self-consumption: The interplay of autophagy and apoptosis. Nat. Rev. Mol. Cell Biol..

[30] Amir M., Zhao E., Fontana L., Rosenberg H., Tanaka K., Gao G., Czaja M.J. (2013). Inhibition of hepatocyte autophagy increases tumor necrosis factor-dependent liver injury by promoting caspase-8 activation. Cell Death Differ..

[31] Chun Y., Kim J. (2018). Autophagy: An Essential Degradation Program for Cellular Homeostasis and Life. Cells.

[32] Chang H., Zou Z. (2020). Targeting autophagy to overcome drug resistance: Further developments. J. Hematol. Oncol..

[33] Sui X., Chen R., Wang Z., Huang Z., Kong N., Zhang M., Han W., Lou F., Yang J., Zhang Q. (2013). Autophagy and chemotherapy resistance: A promising therapeutic target for cancer treatment. Cell Death Dis..

[34] Sun Y., Liu J.H., Jin L., Lin S.M., Yang Y., Sui Y.X., Shi H. (2010). Over-expression of the Beclin1 gene upregulates chemosensitivity to anti-cancer drugs by enhancing therapy-induced apoptosis in cervix squamous carcinoma CaSki cells. Cancer Lett..

[35] Rubinstein A.D., Eisenstein M., Ber Y., Bialik S., Kimchi A. (2011). The autophagy protein Atg12 associates with antiapoptotic Bcl-2 family members to promote mitochondrial apoptosis. Mol. Cell.

[36] Yousefi S., Perozzo R., Schmid I., Ziemiecki A., Schaffner T., Scapozza L., Brunner T., Simon H.U. (2006). Calpain-mediated cleavage of Atg5 switches autophagy to apoptosis. Nat. Cell Biol..

[37] Ciolczyk-Wierzbicka D., Zarzycka M., Gil D., Laidler P. (2019). mTOR inhibitor Everolimus-induced apoptosis in melanoma cells. J. Cell Commun. Signal..

[38] Seo S.U., Woo S.M., Lee H.S., Kim S.H., Min K.J., Kwon T.K. (2018). mTORC1/2 inhibitor and curcumin induce apoptosis through lysosomal membrane permeabilization-mediated autophagy. Oncogene.

[39] Wong V.K.W., Zeng W., Chen J., Yao X.J., Leung E.L.H., Wang Q.Q., Chiu P., Ko B.C.B., Law B.Y.K. (2017). Tetrandrine, an Activator of Autophagy, Induces Autophagic Cell Death via PKC-alpha Inhibition and mTOR-Dependent Mechanisms. Front. Pharm..

[40] Chan E.Y. (2009). mTORC1 phosphorylates the ULK1-mAtg13-FIP200 autophagy regulatory complex. Sci. Signal..

[41] Egan D., Kim J., Shaw R.J., Guan K.L. (2011). The autophagy initiating kinase ULK1 is regulated via opposing phosphorylation by AMPK and mTOR. Autophagy.

[42] Bjorkoy G., Lamark T., Pankiv S., Overvatn A., Brech A., Johansen T. (2009). Monitoring autophagic degradation of p62/SQSTM1. Methods Enzymol..

[43] Wu Y., Jin Y., Sun T., Zhu P., Li J., Zhang Q., Wang X., Jiang J., Chen G., Zhao X. (2020). p62/SQSTM1 accumulation due to degradation inhibition and transcriptional activation plays a critical role in silica nanoparticle-induced airway inflammation via NF-kappaB activation. J. Nanobiotechnol..

[44] Wang H., Mannava S., Grachtchouk V., Zhuang D., Soengas M.S., Gudkov A.V., Prochownik E.V., Nikiforov M.A. (2008). c-Myc depletion inhibits proliferation of human tumor cells at various stages of the cell cycle. Oncogene.

[45] Wang J., Wang H., Li Z., Wu Q., Lathia J.D., McLendon R.E., Hjelmeland A.B., Rich J.N. (2008). c-Myc is required for maintenance of glioma cancer stem cells. PLoS ONE.

[46] Ashwood V.A., Field M.J., Horwell D.C., Julien-Larose C., Lewthwaite R.A., McCleary S., Pritchard M.C., Raphy J., Singh L. (2001). Utilization of an Intramolecular Hydrogen Bond To Increase the CNS Penetration of an NK1 Receptor Antagonist. J. Med. Chem..

[47] Kuhn B., Mohr P., Stahl M. (2010). Intramolecular hydrogen bonding in medicinal chemistry. J. Med. Chem..

[48] Han L., Zhang K., Ishida H., Froimowicz P. (2017). Study of the Effects of Intramolecular and Intermolecular Hydrogen-Bonding Systems on the Polymerization of Amide-Containing Benzoxazines. Macromol. Chem. Phys..

[49] Redza-Dutordoir M., Averill-Bates D.A. (2016). Activation of apoptosis signalling pathways by reactive oxygen species. Biochim. Biophys. Acta.

[50] Wattanathana W., Nootsuwan N., Veranitisagul C., Koonsaeng N., Suramitr S., Laobuthee A. (2016). Crystallographic, spectroscopic (FT-IR/FT-Raman) and computational (DFT/B3LYP) studies on 4,4′-diethyl-2,2′-[methylazanediylbis(methylene)]diphenol. J. Mol. Struct..

[51] Chirachanchai S., Laobuthee A., Phongtamrug S. (2009). Self termination of ring opening reaction of p-substituted phenol-based benzoxazines: An obstructive effect via intramolecular hydrogen bond. J. Heterocycl. Chem..

[52] Wattanathana W., Suetrong N., Kongsamai P., Chansaenpak K., Chuanopparat N., Hanlumyuang Y., Kanjanaboos P., Wannapaiboon S. (2021). Crystallographic and Spectroscopic Investigations on Oxidative Coordination in the Heteroleptic Mononuclear Complex of Cerium and Benzoxazine Dimer. Molecules.

[53] Veranitisagul C., Kaewvilai A., Sangngern S., Wattanathana W., Suramitr S., Koonsaeng N., Laobuthee A. (2011). Novel recovery of nano-structured ceria (CeO(2)) from Ce(III)-benzoxazine dimer complexes via thermal decomposition. Int. J. Mol. Sci..

[54] Kaewvilai A., Rujitanapanich S., Wattanathana W., Veranitisagul C., Suramitr S., Koonsaeng N., Laobuthee A. (2012). The effect of alkali and Ce(III) ions on the response properties of benzoxazine supramolecules prepared via molecular assembly. Molecules.

[55] Wattanathana W., Nonthaglin S., Veranitisagul C., Koonsaeng N., Laobuthee A. (2014). Crystal structure and novel solid-state fluorescence behavior of the model benzoxazine monomer: 3,4-Dihydro-3,6-dimethyl-1,3,2H-benzoxazine. J. Mol. Struct..

