# Supplementary material for: 6,6′-((Methylazanedyl)bis(methylene))bis(2,4-dimethylphenol) Induces Autophagic Associated Cell Death through mTOR-Mediated Autophagy in Lung Cancer
Source: Molecules. 2022 Sep 22;27(19):6230. doi: 10.3390/molecules27196230 (PMC9572635; doi:10.3390/molecules27196230)
Supplement: Supplementary file 1 [file molecules-27-06230-s001.zip › molecules-1881430-supplementary.pdf]

Supporting Information for

6,6′-((methylazanedyl) bis (methylene)) bis (2,4-dimethylphenol) Induces Autophagic Associated Cell Death Through mTOR-mediated Autophagy in Lung Cancer

Nicharat Sriratanasak[1,2], Worawat Wattanathana[3], Pithi Chanvorachote[1,2,*]

[1]Department of Pharmacology and Physiology, Faculty of Pharmaceutical Sciences, Bangkok 10330, Thailand

[2]Center of Excellence in Cancer Cell and Molecular Biology, Faculty of Pharmaceutical Sciences, Chulalongkorn University, Bangkok 10330, Thailand

[3]Department of Materials Engineering, Faculty of Engineering, Kasetsart University, Ladyao, Chatuchak, Bangkok, Thailand

*Correspondence: pithi.c@chula.ac.th.

Spectral data for the synthesized organic compounds

(I) 2,2'-(methylazanediyl)bis(methylene)bis(4-ethylphenol) or EMD

FTIR (cm^{-1}): 3263 (*br*, O–H), 1497 (*vs*, C_a–C_a), 1454 (*m*, N–CH_3), 1272 (*m*, C–O), 1204 (*m*, C–N–C), 820 (*m*, C–N–C); ^{1}H NMR (δ_H, ppm): 1.29 (*d*, *J* = 8.0 Hz, 6H), 2.36 (*s*, 3H), 2.64 (*d*, *J* = 7.5 Hz, 4H), 3.81 (*s*, 4H), 6.85 (*d*, *J* = 8.0 Hz, 2H), 6.99 (*d*, *J* = 2.2 Hz, 2H), 7.05 (*dd*, *J* = 2.0, 8.0 Hz, 2H); ^{13}C NMR (δ_C, ppm): 15.89 (–CH_3), 27.94 (–CH_2–C_a), 40.98 (CH_3–NR_2), 59.24 (–CH_2–NR_2), 115.80 (C_a), 122.31 (C_a), 128.22 (C_a), 129.47 (C_a), 135.16 (C_a), 154.19 (C–OH). (Yield: 89%)

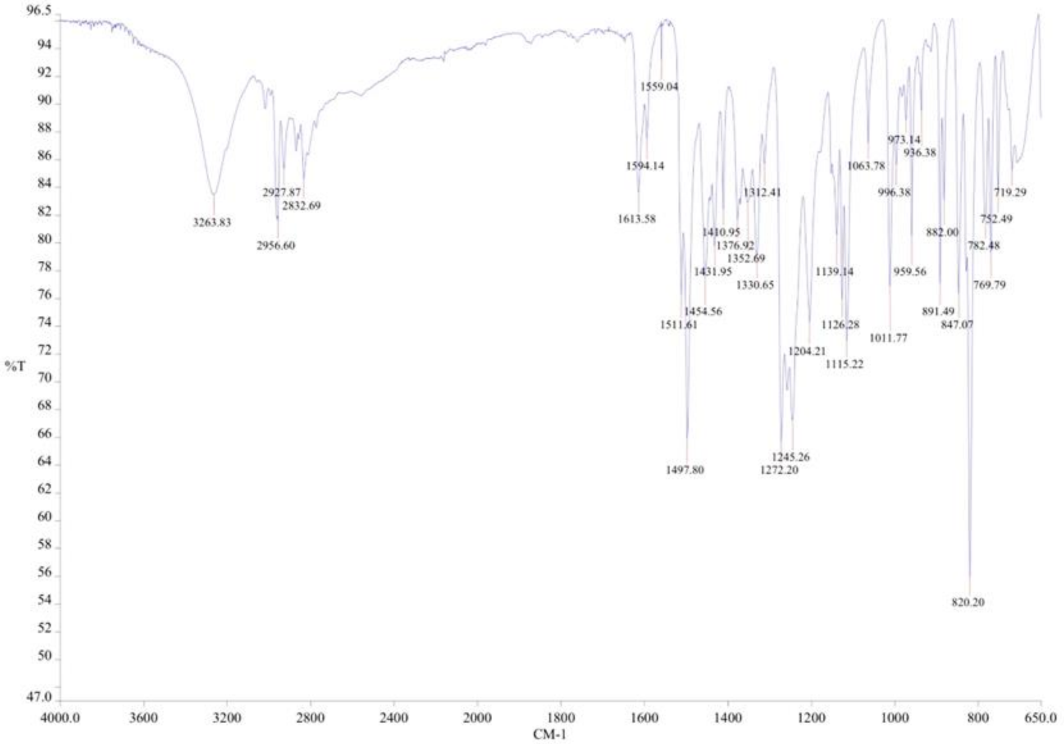

Figure S1 Fourier-transform infrared spectrum of EMD

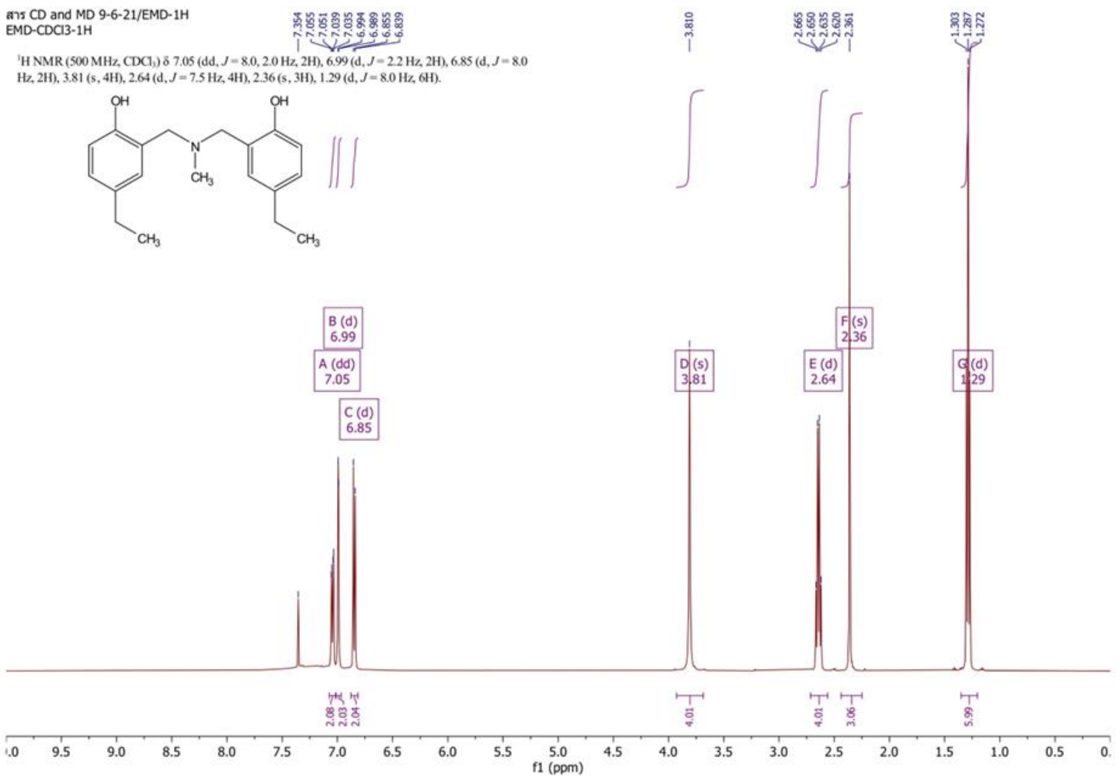

Figure S2 ¹H-NMR spectrum of EMD

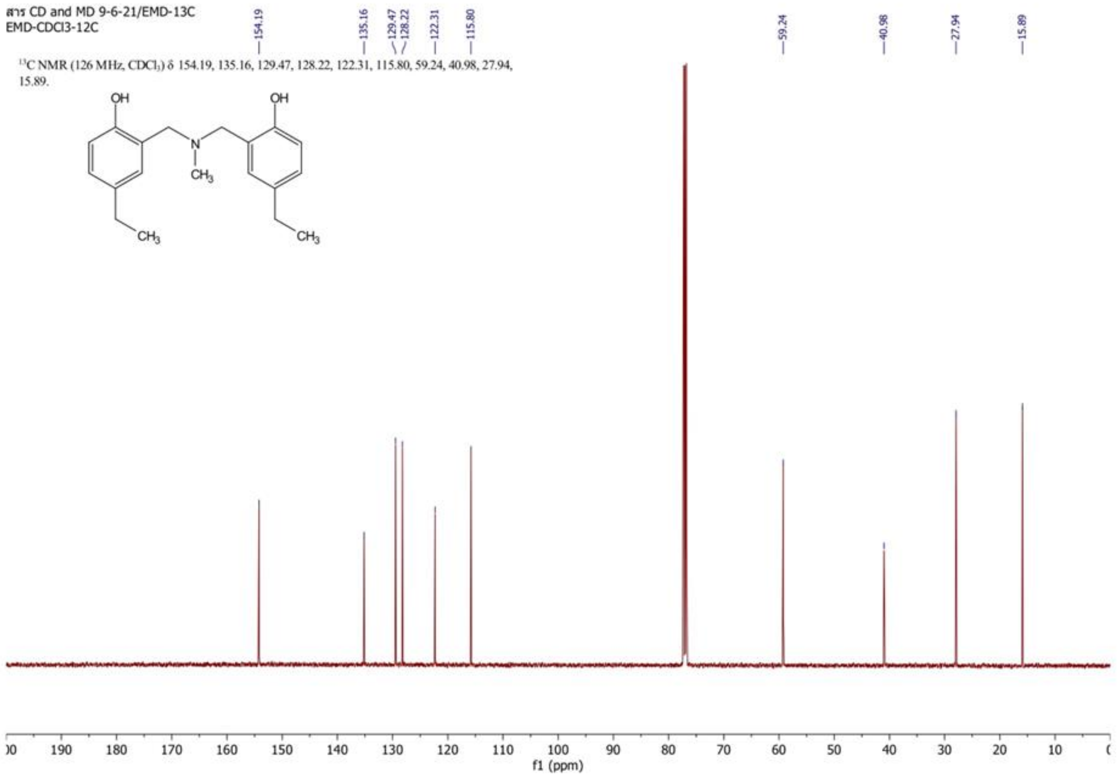

Figure S3 ¹³C-NMR spectrum of EMD

(II) 6,6'-(methylazanediyl)bis(methylene) 409 bis(2,4-dimethylphenol) or 24MD

FTIR (cm^{-1}): 3391 (*br*, O–H), 1482 (*vs*, C_a–C_a), 1456 (*m*, N–CH_3), 1241 (*m*, C–O), 1199 (*m*, C–N–C), 846 (*m*, C–N–C); ^{1}H NMR (δ_H, ppm): 2.32 (*s*, 12H), 2.34 (*s*, 3H), 3.77 (*s*, 4H), 6.83 (*d*, *J* = 2.5 Hz, 2H), 6.98 (*d*, *J* = 2.5 Hz, 2H); ^{13}C NMR (δ_C, ppm): 15.86 (–CH_3), 20.42 (–CH_3), 41.06 (CH_3–NR_2), 59.35 (–CH_2–NR_2), 121.41 (C_a), 124.54 (C_a), 128.40 (C_a), 128.59 (C_a), 131.15 (C_a), 151.95 (C–OH). (Yield: 80%)

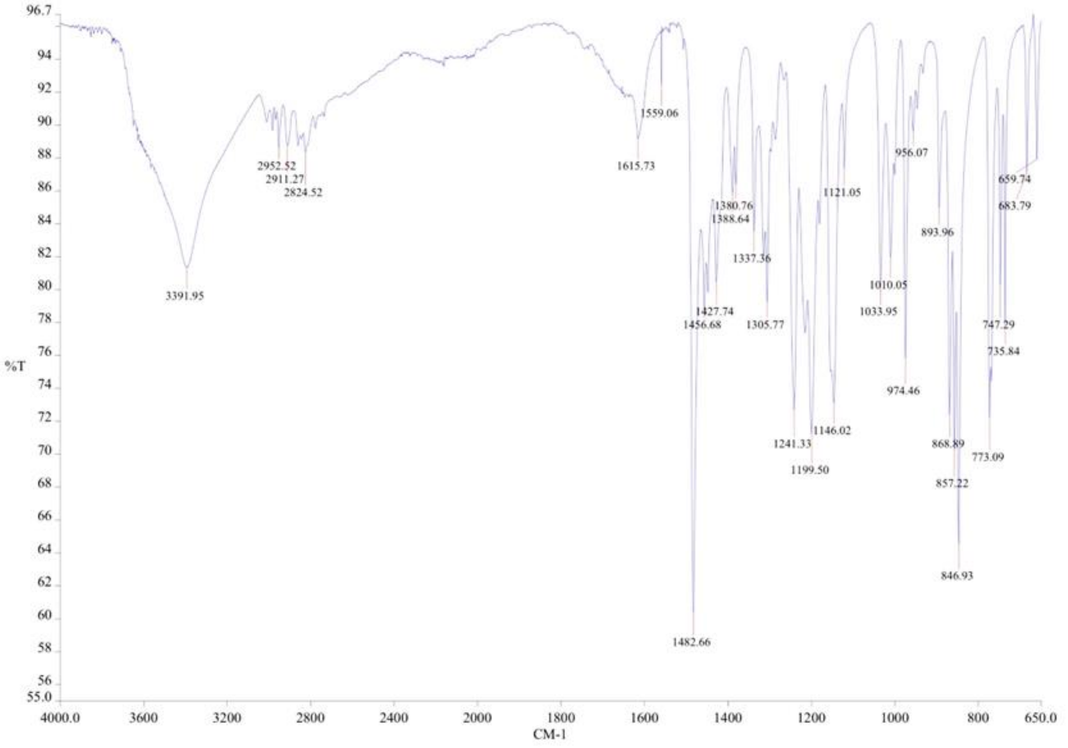

Figure S4 Fourier-transform infrared spectrum of 24MD

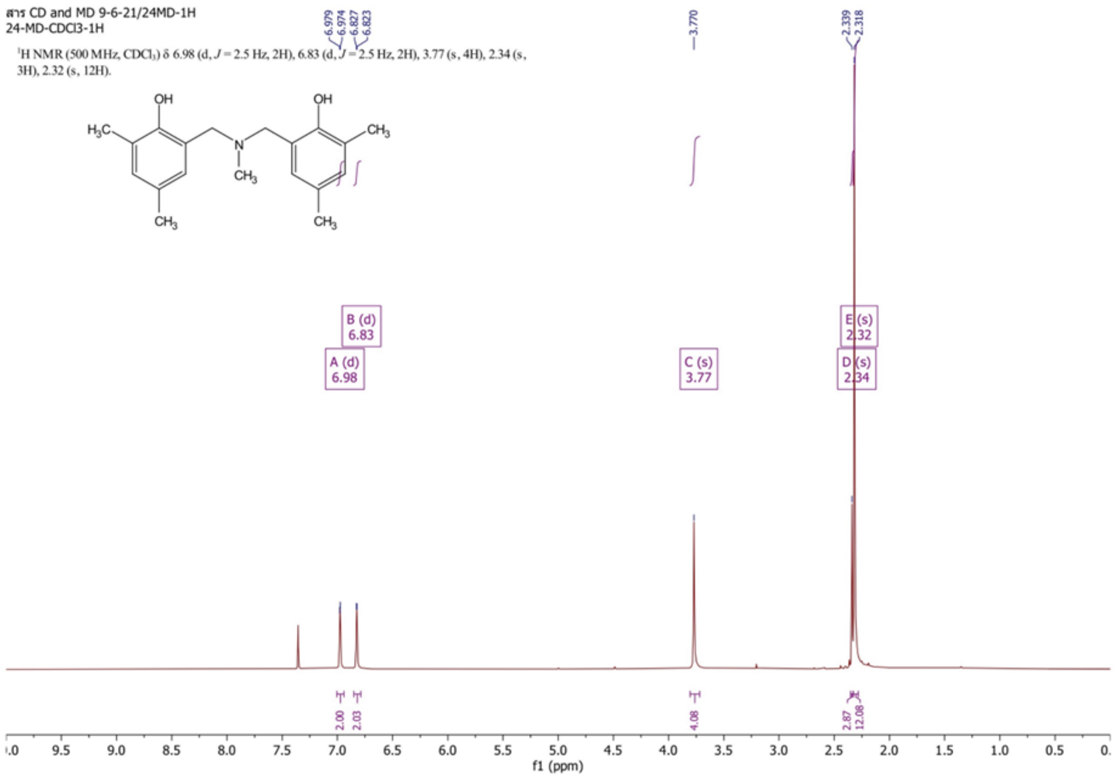

Figure S5 ^{1}H-NMR spectrum of 24MD

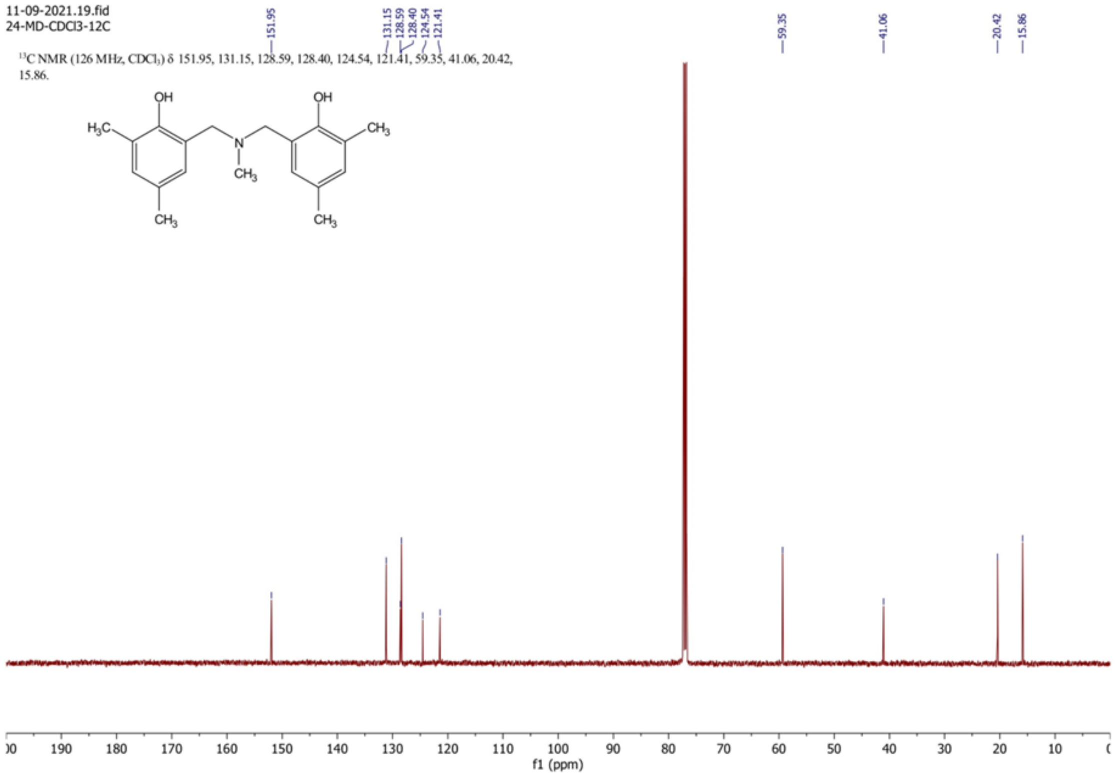

Figure S6 ^{13}C-NMR spectrum of 24MD

(III) 6,6'-(cyclohexylazanediyl)bis(methylene) 410 bis(2,4-dimethylphenol) or 24CD

FTIR (cm^{-1}): 3452 (*br*, O–H), 1484 (*vs*, C_a–C_a), 1451 (*m*, N–C_{cy}), 1171 (*m*, C–O), 1153 (*m*, C–N–C), 854 (*m*, C–N–C); ^{1}H NMR (δ_H, ppm): 1.14–1.23 (*m*, 1H), 1.24–1.34 (*m*, 2H), 1.46–1.61 (*m*, 2H), 1.73 (*dt*, *J* = 3.5, 13.0 Hz, 1H), 1.91 (*dt*, *J* = 3.5, 13.0 Hz, 2H), 2.03 (*dd*, *J* = 3.5, 9.0 Hz, 2H), 2.30 (*d*, *J* = 4.5 Hz, 12H), 2.81 (*tt*, *J* = 3.5, 12.0 Hz, 1H), 3.83 (*s*, 4H), 6.79 (*d*, *J* = 2.0 Hz, 2H), 6.95 (*d*, *J* = 2.0 Hz, 2H); ^{13}C NMR (δ_C, ppm): 15.90 (–CH_3), 20.45 (C_{cy}), 25.78 (–CH_3), 26.15 (C_{cy}), 27.45 (C_{cy}), 51.48 (–CH_2–NR_2), 57.43 (C_{cy}–NR_2), 121.69 (C_a), 124.83 (C_a), 128.45 (C_a), 128.50 (C_a), 130.90 (C_a), 152.06. (Yield: 89%)

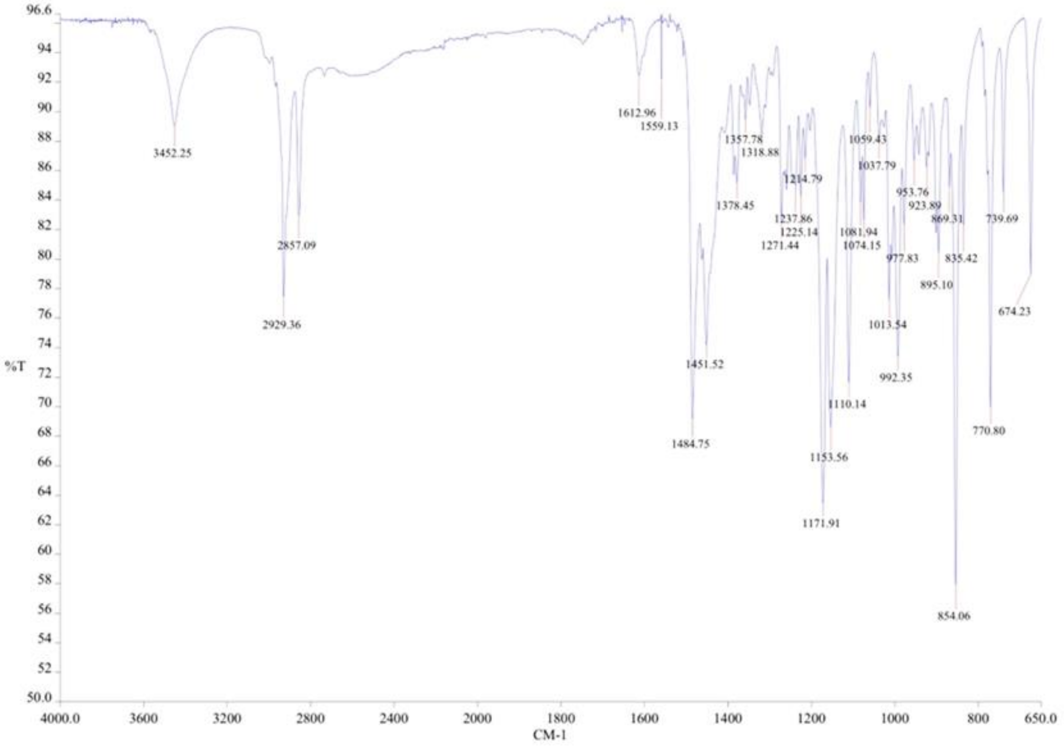

Figure S7 Fourier-transform infrared spectrum of 24CD

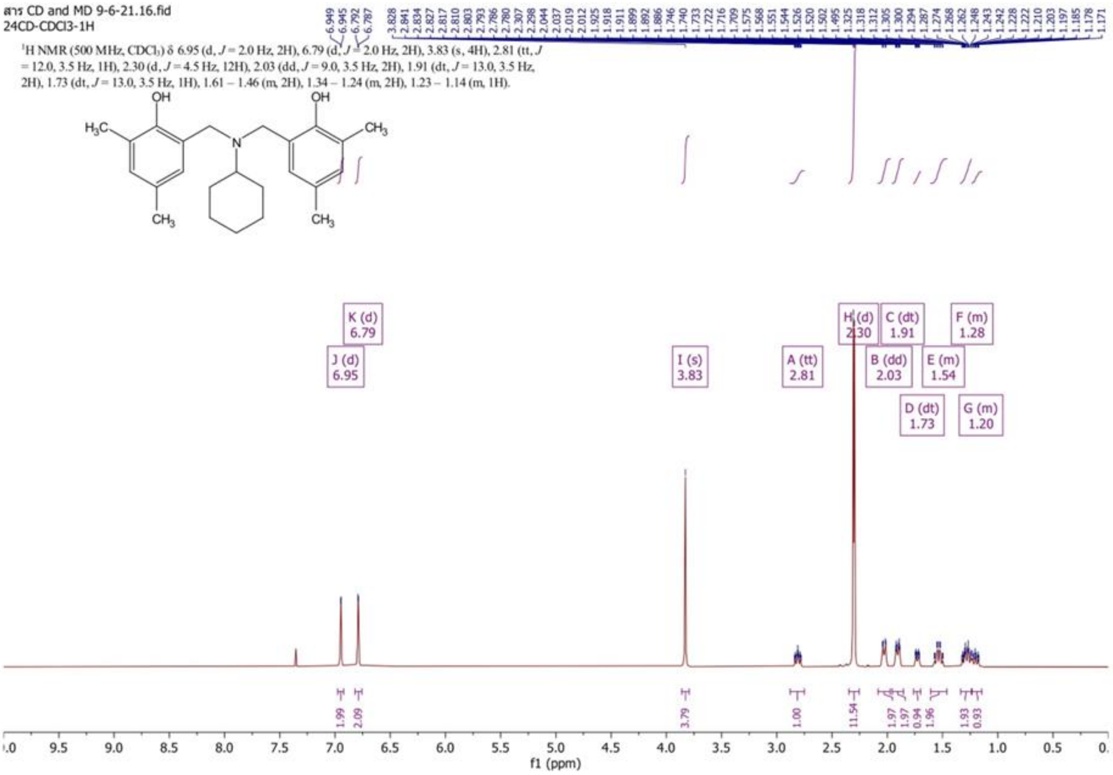

Figure S8 ^{1}H-NMR spectrum of 24CD

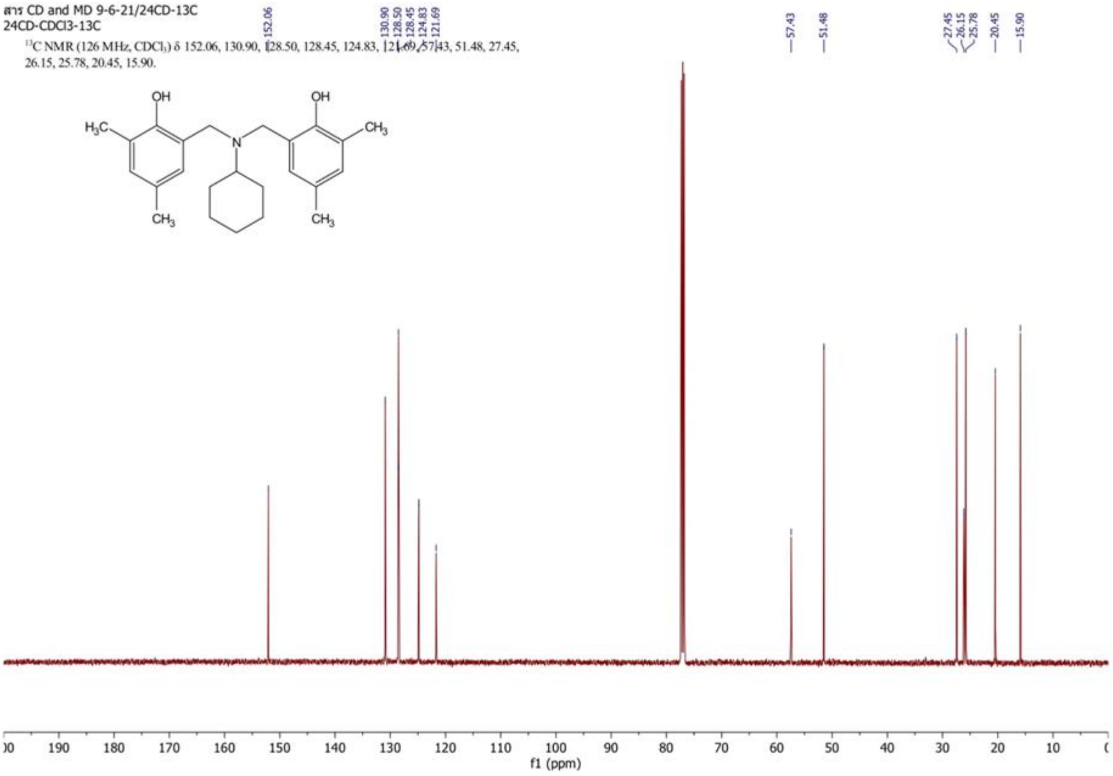

Figure S9 ^{13}C-NMR spectrum of 24CD
